# Lack of Association Between Polymorphisms in *TXNRD2* and *LMX1B* and Primary Open-Angle Glaucoma in a Saudi Cohort

**DOI:** 10.3389/fgene.2021.690780

**Published:** 2021-08-02

**Authors:** Altaf A. Kondkar, Taif A. Azad, Abdullah S. Alobaidan, Tahira Sultan, Essam A. Osman, Faisal A. Almobarak, Glenn P. Lobo, Saleh A. Al-Obeidan

**Affiliations:** ^1^Department of Ophthalmology, College of Medicine, King Saud University, Riyadh, Saudi Arabia; ^2^Glaucoma Research Chair in Ophthalmology, College of Medicine, King Saud University, Riyadh, Saudi Arabia; ^3^King Saud University Medical City, King Saud University, Riyadh, Saudi Arabia; ^4^College of Medicine, King Saud University, Riyadh, Saudi Arabia; ^5^Department of Ophthalmology and Visual Neurosciences, University of Minnesota, Minneapolis, MN, United States

**Keywords:** genetics, glaucoma, intraocular pressure, mitochondrial dysfunction, oxidative stress, rs35934224, rs6478746

## Abstract

**Objective:** Recent studies have demonstrated an association of single nucleotide polymorphisms (SNPs) rs35934224 in *TXNRD2* and rs6478746 near *LMX1B* genes in primary open-angle glaucoma (POAG) among Europeans. We performed a retrospective, case-control study to investigate the association between the rs35934224 (*TXNRD2*) and rs6478746 (*LMX1B*) and POAG in a middle-eastern population from Saudi Arabia.

**Methods:** DNA from 399 participants consisting of 150 POAG cases (83 males and 67 females) and 249 controls (135 males and 114 females) were genotyped using TaqMan® real-time PCR. Statistical tests were performed to evaluate genetic association with POAG and related clinical indices.

**Results:** The minor allele frequency (MAF) of rs35934224[T] was 0.19 and 0.20 in POAG and controls, respectively. The difference was non-significant (odds ratio [OR] = 1.08, 95% confidence interval [CI] = 0.75–1.55, *p* = 0.663). Likewise, rs6478746[G] MAF was 0.12 in both cases and controls with no statistical significance (OR = 1.02, 95% CI = 0.67–1.56, *p* = 0.910). Genotype analysis showed no association with POAG for both the SNPs in combined and gender-stratified groups. Regression analysis showed no significant effect of risk factors such as age, sex, rs35934224, and rs6478746 genotypes on POAG outcome. Furthermore, both the SNPs showed no significant genotype effect on clinical indices such as intraocular pressure (IOP) and cup/disc ratio in POAG patients.

**Conclusions:** Rs35934224 in *TXNRD2* and rs6478746 near *LMX1B* genes are not associated with POAG or related clinical indices such as IOP and cup/disc ratio in a Saudi cohort. Since the study is limited by sample size further investigations are needed to confirm these results in a larger cohort.

## Introduction

Primary open-angle glaucoma (POAG) is among the most common subtype of glaucoma worldwide, including the middle-east (Al Obeidan et al., 2011). Several genetic and hereditary factors such as family history, age, intraocular pressure (IOP), and ancestry are established risk factors of POAG (Abu-Amero et al., 2015; Kreft et al., 2019). The ethnic predisposition combined with the familial tendency strongly supports the involvement of genetic components in the pathogenesis of POAG (Abu-Amero et al., 2015). In the recent past, genome-wide association studies (GWAS) have identified several significant associations of POAG or its endophenotypic traits with single nucleotide polymorphisms (SNPs) in different genes/loci (Abu-Amero et al., 2015; Trivli et al., 2020). These include the rs35934224 in the thioredoxin reductase 2 (*TXNRD2*) genomic region (Bailey et al., 2016), and rs6478746 on chromosome 9 near LIM homeobox transcription factor 1 beta (*LMX1B*) and an uncharacterized RNA gene, *LOC105376277* identified among the POAG patients of European ancestry (Gharahkhani et al., 2018).

Rs35934224 is located in *TXNRD2* gene encoding a nuclear-encoded mitochondrial protein that plays a crucial role in regulating cellular redox homeostasis, mainly by scavenging reactive oxygen species (ROS) in the mitochondria. There is increasing evidence for the role of oxidative stress and mitochondrial dysfunction in POAG susceptibility (Lee et al., 2011; Chrysostomou et al., 2013; Khawaja et al., 2016). Interestingly, previous studies have pointed out to a strong role of oxidative stress mechanisms and mitochondrial dysfunction in middle-eastern POAG patients making this gene/variant an important candidate to investigate in this population (Abu-Amero et al., 2015). Furthermore, oxidative stress has also been reported to induce retinal ganglion cell (RGC) dysfunction in glaucoma (Chrysostomou et al., 2013). Increased expression of thioredoxins 1 and 2 (the substrate of thioredoxin reductases) was found to prolong RGC survival in experimental glaucoma (Caprioli et al., 2009). *TNXRD2* is also a stress response gene involved in many signaling pathways, including Wnt signaling, which plays a significant role in glaucoma pathogenesis (Kipp et al., 2012; Dhamodaran et al., 2020).

The variant rs6478746 is located in *LMX1B* that codes for a transcription factor and has a role in normal development. Mutations in *LMX1B* are associated with the nail-patella syndrome, in which some patients also develop glaucoma as one of the manifestations of the syndrome (Mimiwati et al., 2006). Animal studies have also provided further evidence for the role of *Lmx1b* in glaucoma (Cross et al., 2014). This gene plays an essential role in morphogenesis of the anterior segment of the eye and IOP regulation (Liu and Johnson, 2010). *LMX1B* was reported to be highly expressed in the trabecular meshowork (TM) and the variant rs6478746 within this locus was suggested to alter its expression (Gharahkhani et al., 2018), and may thereby influence POAG outcomes either via interference in the developmental processes or through IOP regulation.

The genetic basis of POAG in patients of Saudi origin is still largely unknown with many studies in the past reporting conflicting association of genetic variants reported among Caucasians/Asians in this ethnicity (Abu-Amero et al., 2016; Kondkar et al., 2016, 2017, 2018a,b, 2019), and therefore warrants further genetic analysis. To identify a genetic link and provide further validation of POAG-associated variants in this ethnic group, we performed a retrospective, case-control study to investigate the association between polymorphisms rs35934224 and rs6478746 in *TXNDR2* and *LMX1B*, respectively and POAG in a Saudi cohort.

## Methods

### Study Design and Participants

A retrospective case-control study was performed in 150 unrelated Saudi patients with POAG and 249 control subjects. The study adhered to the Declaration of Helsinki principles and was approved by the institutional review board committee at the College of Medicine, King Saud University, Riyadh, Saudi Arabia. All the participants signed informed consent. The clinical criteria for patients' inclusion were as described previously (Abu-Amero et al., 2018). Each patient underwent a standardized ophthalmic examination by glaucoma specialists at the ophthalmology screening clinics of King Abdulaziz University Hospital. Briefly, the clinical diagnosis was based on (i) the presence of glaucomatous optic disk damage or retinal nerve fiber layer changes; (ii) presence of visual field abnormalities without any other cause(s) or explanation; (iii) open anterior chamber angle; and (iv) adult-onset. Patients having secondary glaucoma, trauma, or on steroids were excluded. Non-glaucomatous individuals with normal IOP (<21 mmHg without any medication) on examination and >40 years of age served as controls. Individuals refusing to participate were excluded.

### Genotyping of rs35934224 and rs6478746

DNA samples were genotyped using TaqMan® assays, C___2539086_10 and C___1440530_10 (Catalog number: 4351379; Applied Biosystems Inc., Foster City, CA, USA) for SNPs rs2472493 and rs7636836, respectively, on ABI 7500 Real-Time PCR System (Applied Biosystems) according to the manufacturer instructions under recommended amplification conditions (Abu-Amero et al., 2013a).

### Statistical Analyses

Statistical tests were performed using SPSS version 22 (IBM Inc., Chicago, Illinois, USA). Student's *t*-test (2-groups) and one-way ANOVA (3-groups) were used to analyze continuous variables. SNPStats online software (https://www.snpstats.net/start.htm) was used for testing genotype associations. Binary logistic regression analysis was performed to test the effects of multiple risk factors such as age, sex, and genotypes on disease outcomes. Power analysis was done using an open-source online PS program version 3.1.2 for unmatched case-control (dichotomous) testing (https://vbiostatps.app.vumc.org/ps/). A *p* < 0.05 (2-tailed) was considered significant. Bonferroni's correction was used to adjust for multiple testing, and corrected *p* < 0.025 were considered where applicable.

## Results

### Demographic Data

A total of 399 individuals consisting of 150 cases with POAG (83 males and 67 females) and 249 controls (135 males and 115 females) were included. The mean age was 61.3 (± 10.1) years for cases and 59.7 (± 7.0) years for controls. The two study groups were similar for age (*p* = 0.071) and gender (*p* = 0.828) distribution with no statistical significance (*p* = 0.071 and 0.828, respectively) (Supplementary Figure 1).

### Allele Frequency of rs35934224 and rs6478746 in POAG

Table 1 shows the allele frequency distribution of rs35934224 and rs6478746 in POAG and according to gender in cases and controls. The control group showed no significant deviation from Hardy-Weinberg equilibrium (*p* > 0.05). The minor allele frequency (MAF) distribution showed no significant association with POAG for both polymorphisms. Likewise, gender-stratification also showed no significant association.

**Table 1:** Minor allele frequencies of rs35934224 (*TXNRD2*) and rs6478746 (*LMX1B*) in POAG cases and controls.

**SNP Group**	**Controls MAF**	**POAG MAF**	**Odds ratio (95% confidence interval)**	***p*-value**
**Rs35934224[T]**				
Total	0.19	0.20	1.08 (0.75–1.55)	0.663
Men	0.17	0.22	1.36 (0.84–2.20)	0.208
Women	0.21	0.19	0.90 (0.52-1.55)	0.700
**Rs6478746[G]**				
Total	0.12	0.12	1.02 (0.67–1.56)	0.910
Men	0.13	0.13	1.00 (0.57–1.77)	0.990
Women	0.11	0.12	1.05 (0.56–1.95)	0.890

*POAG, primary open-angle; MAF, minor allele frequency*.

*p-value determined by Pearson's Chi-square analysis*.

### Genotype Analysis of rs35934224 and rs6478746 in POAG

Genotype analysis was performed using SNPStats software in different genetic models, as shown in Table 2. Both rs35934224 and rs6478746 genotypes did not show any significant association with POAG. However, the T/T genotype presence was found to increase the risk of POAG by more than 2-fold (OR = 2.62) compared to the C/C genotype, but the effect did not achieve statistical significance (*p* = 0.088). Furthermore, genotype analysis of both the variants based on gender stratification did not show any gender-specific association with POAG in any of the tested genetic models than controls (Supplementary Tables 1, 2).

**Table 2:** Association analysis of rs35934224 in *TXNRD2* and rs6478746 in *LMX1B* with primary open-angle glaucoma.

**Gene SNP**	**Genetic model**	**Genotype**	**Control n (%)**	**POAG n (%)**	**Odds ratio (95% confidence interval)**	***p*-value**	*p*-value^*^
*TXNRD2* rs35934224	Codominant	C/C	159 (63.9)	97 (64.7)	1.00	0.170	0.150
		C/T	85 (34.1)	45 (30.0)	0.87 (0.56–1.35)		
		T/T	5 (2.0)	8 (5.3)	2.62 (0.83–8.25)		
	Dominant	C/C	159 (63.9)	97 (64.7)	1.00	0.870	0.760
		C/T-T/T	90 (36.1)	53 (35.3)	0.97 (0.63–1.47)		
	Recessive	C/C-C/T	244 (98.0)	142 (94.7)	1.00	0.076	0.076
		T/T	5 (2.0)	8 (5.3)	2.75 (0.88–8.57)		
*LMX1B* rs6478746	Codominant	A/A	195 (78.3)	117 (78.0)	1.00	0.990	0.930
		A/G	48 (19.3)	29 (19.3)	1.01 (0.60–1.68)		
		G/G	6 (2.4)	4 (2.7)	1.11 (0.31–4.02)		
	Dominant	A/A	195 (78.3)	117 (78.0)	1.00	0.940	0.730
		A/G-G/G	54 (21.7)	33 (22.0)	1.02 (0.62–1.66)		
	Recessive	A/A-A/G	243 (97.6)	146 (97.3)	1.00	0.870	0.990
		G/G	6 (2.4)	4 (2.7)	1.11 (0.31–4.00)		

* *p-value adjusted for age and sex*.

*POAG, primary open-angle glaucoma; TXNRD2, thioredoxin reductase 2; LMX1B, LIM homeobox transcription factor 1 beta*.

### Effect of Age, Sex, and Genotypes on POAG Outcome

Table 3 shows the binary logistic regression analysis of age, sex, rs35934224, and rs6478746 variants on the disease outcome. None of these risk factors showed any significant effect on POAG outcome.

**Table 3:** Binary logistic regression analysis to determine the effect of age, sex, and polymorphisms on POAG risk.

**Group variables**	**B**	**SE**	**Wald**	**Odds ratio (95% confidence interval)**	***p*-value**
Age	0.023	0.013	3.343	1.02 (0.99–1.05)	0.067
Sex	0.010	0.210	0.002	1.01 (0.67–1.52)	0.962
Rs35934224			3.485		0.175
C/T	−0.148	0.226	0.429	0.862 (0.55–1.34)	0.512
T/T	0.972	0.588	2.738	2.64 (0.83–8.37)	0.098
Rs6478746			0.138		0.934
A/G	0.087	0.268	0.107	1.09 (0.64–1.84)	0.744
G/G	0.133	0.664	0.040	1.14 (0.31–4.19)	0.841
Constant	−1.929	0.798	5.848	0.145	0.016

*POAG, primary open-angle glaucoma*.

### Effect on Genotypes on Clinical Indices of POAG

**Figure 1:**
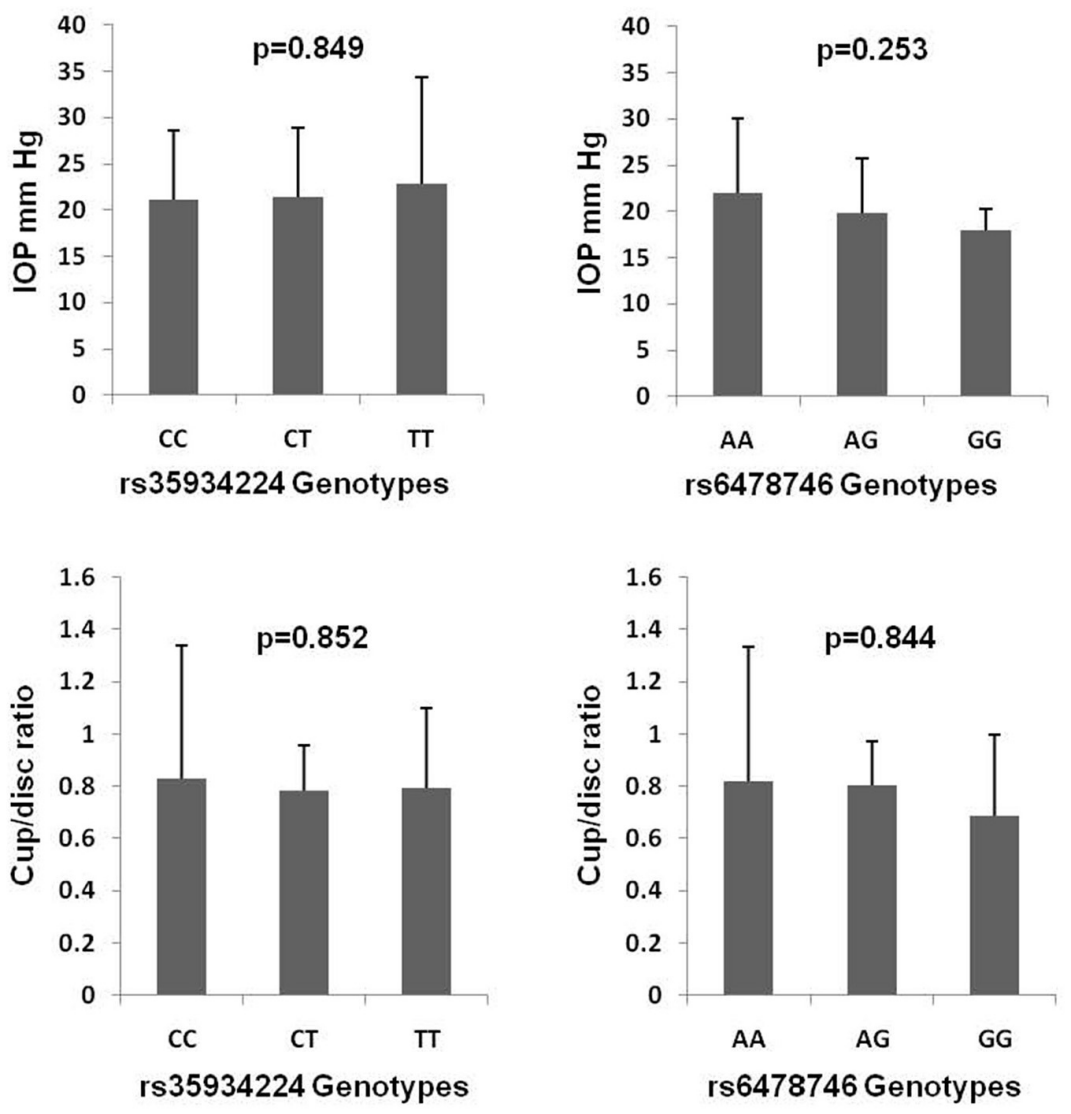
Genotype effects of rs35934224 and rs6478746 variants on intraocular pressure (IOP) and cup/disc ratio in primary open-angle glaucoma. *p*-values are based on one-way ANOVA analysis.

Figure 1 shows the genotype effect of rs35934224 and rs6478746 variants on IOP and cup/disc ratio in POAG. The analysis revealed no significant genotype effect on these clinical phenotypes that reflect disease severity in glaucoma patients.

## Discussion

POAG follows a complex multifactorial inheritance pattern involving genetics and environmental factors (Abu-Amero et al., 2015). Despite a high burden of POAG in the Saudi population, specific genetic markers and their influence on disease pathogenesis are still not well-defined. With several polymorphisms reported to be associated with POAG or related endophenotypic traits (Abu-Amero et al., 2015), we investigated whether SNPs rs35934224 (*TXNRD2*) and rs6478746 (*LMX1B*) are associated with POAG in the middle-eastern cohort of Saudi Arabia and report a negative association of these variants with POAG in this ethnic group.

In a meta-analysis of GWAS findings of individuals of European descent from the United States, referred to as National Eye Institute Glaucoma Human Genetics Collaboration Heritable Overall Operational Database (NEIGHBORHOOD), and replication in an Australian and New Zealand Registry of Advanced Glaucoma (ANZRAG) study, Blue Mountains Eye Study (BMES) (second study from Australia), 3 European studies from European Prospective Investigation into Cancer-Norfolk Eye study (EPIC), Germany, United Kingdom (UK), and a Singaporean Chinese dataset, Bailey et al. (2016) identified novel loci, including rs35934224[T] within *TXNRD2* exhibiting a significant protective effect in POAG (OR = 0.78, *p* = 4.05 × 10^−11^).

The rs35934224[T] MAF in our Saudi POAG patients (0.20) was found to be higher than those reported in the POAG patients of European descent (0.13), the Singaporean Chinese (0.02) (Bailey et al., 2016), and the Japanese (0.05) (Shiga et al., 2018), but much lower than POAG patients in the Genetics In Glaucoma patients from African descent (GIGA) study (0.31) (Bonnemaijer et al., 2018). Unlike Bailey et al., we did not observe any allelic or genotype effect of this SNP in POAG in the Saudi cohort. Although the meta-analysis (Bailey et al., 2016) reported a positive association with POAG, the individual analysis showed no association among Germany, UK, BMES, and Singaporean Chinese, with a modest association (*p* = 0.03) in the EPIC cohorts. Similarly, rs35934224 in *TXNRD2* also showed no association among the Japanese POAG patients (Shiga et al., 2018) and that of the African ancestry (*p* = 0.369) (Bonnemaijer et al., 2018). But, a proxy variant rs16984299[C] in the latter study (Bonnemaijer et al., 2018) was associated with POAG in the same direction as reported by Bailey et al. (OR = 0.83, 95% CI = 0.74–0.94, *p* = 0.0032) that demonstrated a borderline association after adjustment for multiple testing (*p* = 0.049). Besides, rs35934224 was significantly associated with POAG in non-Hispanic Whites (*p* = 2.9 × 10^−5^), but not in the Hispanic Latinos (*p* = 0.72), East Asians (*p* = 0.22), and the African Americans (*p* = 0.60) of the Genetic Epidemiology Research in Adult Health and Aging (GERA) cohort (Choquet et al., 2018). These results suggest that this variant exhibits racial variations and maybe a useful genetic marker among the White population.

*TXNRD2* encodes a selenocysteine-containing mitochondrial protein, a key player in the defense against oxidative damage and which has been demonstrated to be expressed in all the primary human ocular tissues (Bailey et al., 2016). Alterations in TXNRD2 function may plausibly result in mitochondrial abnormalities and increased oxidative stress. Many studies in the past have implied an essential role of mitochondrial abnormalities and oxidative stress in the pathogenesis of POAG, including patients of Saudi origin (Abu-Amero et al., 2006, 2013b; Lascaratos et al., 2012; Kondkar et al., 2018c, 2020). Likewise, mitochondrial dysfunction has also been implicated in RGC loss in experimental glaucoma models (Osborne, 2008; Lascaratos et al., 2012). Although the precise mechanisms are not fully understood, the resulting mitochondrial dysfunction may contribute to energy deficit in the glaucomatous optic nerve and affect the IOP (Coughlin et al., 2015). Besides, long-term accumulation of oxidative damages as a result of mitochondrial and endothelial dysfunction may cause oxidative imbalance and trigger TM apoptosis leading to glaucoma (Sacca et al., 2015). In support, the overexpression of thioredoxin was reported to protect the RGCs from apoptosis after optic nerve axotomy in pharmacologically induced oxidative stress in an *in-vitro* and *in-vivo* animal model of glaucoma (Caprioli et al., 2009). Cheng et al. demonstrated that the neuroprotective effect of pituitary adenylate cyclase-activating polypeptide (PACAP), an endogenous peptide, is associated with the regulation of mitochondrial function in the RGC-5 cells (Cheng et al., 2018).

rs6478746[G] near *LMX1B* was identified to be associated with POAG in the ANZRAG cohort of the European ancestry (*p* = 4.54 × 10^−8^) and replicated in the NEIGHBORHOOD cohort (*p* < 0.0125) (Gharahkhani et al., 2018). In contrast, our results did not show any allele or genotype association of this variant with POAG in the Saudi cohort. Although rs6478746 has not been evaluated in other ethnic groups, different variants in *LMX1B*, such as rs55770306 was associated with POAG in the Genetic Epidemiology Research in Adult Health and Aging (GERA) cohort (*p* = 0.00019) consisting of the non-Hispanic whites, Hispanic/Latinos, East Asians, and African-Americans, and the UK Biobank (UKB) cohort (*p* = 5.5 × 10^−14^) in the same study (Choquet et al., 2018); and rs10819187 was identified in a Japanese GWAS (*p* = 4.4 × 10^−12^) (Shiga et al., 2018) supporting the role of *LMX1B* in POAG.

*LMX1B* mutations have been suggested to contribute to glaucoma resulting from ocular developmental anomalies (Mimiwati et al., 2006; Ghoumid et al., 2016). A mouse model carrying a dominant-negative mutation of *Lmx1b* was demonstrated to develop high IOP, abnormalities of the iridocorneal angle, and abnormal TM, which are all critical risk factors for developing glaucoma in humans (Cross et al., 2014). Besides, specific *Lmx1b* mutations were also recently reported to cause increased IOP and glaucoma resembling POAG phenotype in mice with variable anterior segment developmental anomalies that were dependent on the genetic background (Choquet et al., 2018; Tolman et al., 2021). These observations further strengthened the role of *LMX1B* in the glaucoma concept and suggested that it may affect the risk of glaucoma even in the absence of any obvious developmental defects in the human eye in the general population. *Lmx1b* has also been previously shown to be required to develop the murine TM and IOP regulation (Liu and Johnson, 2010). In support, *LMX1B* was reported to be significantly associated with vertical cup/disc ratio (*p* = 0.0035) and moderately with IOP (*p* = 0.0315) (Gharahkhani et al., 2018). Furthermore, variants in *LMX1B* and *TXNRD2* were also among the 101 novel loci associated with IOP in a GWAS meta-analysis performed by MacGregor et al. (2018). However, the variants rs6478746 in *LMX1B* and rs35934224 in *TXNRD2* did not correlate with IOP and cup/disc ratio in our POAG cohort.

Although recent studies demonstrated an association of SNPs rs35934224 and rs6478746 in *TXNDR2* and *LMX1B*, respectively, in POAG patients of European origin (Bailey et al., 2016; Gharahkhani et al., 2018), our study did not find a similar pathogenic association of these SNPs in POAG patients of Saudi origin. Several factors and certain limitations in this study can explain this disagreement. The prevalence of glaucoma is known to vary with race and is reported to be more prevalent and severe in African ancestry than European descent patients. The racial differences are further complicated by differing allele frequency and effect size of loci. In fact, most of the genetic findings from the European and Asian GWAS studies have not been fully replicated in POAG patients of Saudi origin (Abu-Amero et al., 2015), indicating the possible presence of ethnic-specific variants that remain to be identified or validated (Hauser et al., 2019; Kondkar et al., 2021). Besides race, the clinical differences and sampling variability in the study population may also be responsible for these discrepanices. Ours being a tertiary care center, there could be referral or selection bias. Furthermore, the presence of other variants in these genes, gene-gene or gene-environment interactions influencing the study outcome has not been investigated and hence cannot be ruled out. Lastly, a negative association may be a result of lack of sufficient statistical power to detect significant associations with POAG or its indices (e.g., IOP and cup/disc ratio) in this cohort. The sample size examined in this study is relatively small, with even fewer numbers in subgroup analyses, which contributes to the major limitation of this study. Based on the MAF observed in our study population, assuming an OR of 2.0 with 2-sided test and type I error of 0.05, we had 0.83 and 0.70 probabilities of detecting any significant associations between POAG and rs35934224 and rs6478746, respectively. However, to detect a <1.5-fold relative risk, as is the case with most case-control genetic association studies where SNP(s) exhibit a relatively small effect and also reported by Bailey et al. (2016) and Gharahkhani et al. (2018), or to estimate a clear genotype-phenotype correlation much larger cohort needs to be examined. A large population-based multicenter study in POAG patients of Arab origin would be needed to confirm these possibilities.

In conclusion, our study did not observe any direct association between allele and genotype frequencies of SNPs rs35934224 and rs6478746 in *TXNDR2* and *LMX1B*, respectively, and POAG or its related phenotypes such as IOP and cup/disc ratio, suggesting that these SNPs may not be significant risk factors of POAG in this ethnic group. However, since the study is limited by sample size the results would require cautious interpretation and further investigations to confirm them in a larger cohort. Besides, the role of other variants in these gene in POAG also cannot be ruled out.

## Data Availability Statement

The original contributions presented in the study are included in the article/Supplementary Material, further inquiries can be directed to the corresponding author/s.

## Ethics Statement

The studies involving human participants were reviewed and approved by Institutional review board (IRB) and research ethics committee of the College of Medicine, King Saud University. The patients/participants provided their written informed consent to participate in this study.

## Author Contributions

AK: concept, experimental design, analysis, data interpretations, wrote, and edited the manuscript. TA, AA, and TS: sample preparation, genotyping, data acquisition, and manuscript editing. EO and FA: clinical diagnosis and recruitment, data interpretation, manuscript editing, and revision. GL: data interpretations, manuscript editing, and revision. SA-O: concept, clinical diagnosis and recruitment, data interpretation, manuscript editing, and revision. All authors have read and approved the final manuscript.

## Conflict of Interest

The authors declare that the research was conducted in the absence of any commercial or financial relationships that could be construed as a potential conflict of interest.

## Publisher's Note

All claims expressed in this article are solely those of the authors and do not necessarily represent those of their affiliated organizations, or those of the publisher, the editors and the reviewers. Any product that may be evaluated in this article, or claim that may be made by its manufacturer, is not guaranteed or endorsed by the publisher.
