# Supplementary material for: Lack of Association Between Polymorphisms in TXNRD2 and LMX1B and Primary Open-Angle Glaucoma in a Saudi Cohort
Source: Front Genet. 2021 Aug 2;12:690780. doi: 10.3389/fgene.2021.690780 (PMC8365832; doi:10.3389/fgene.2021.690780)
Supplement: Supplementary file 1 [file Data_Sheet_1.doc]

**Supplementary Fig. S1.** Demographic data distribution of subjects included in this study.


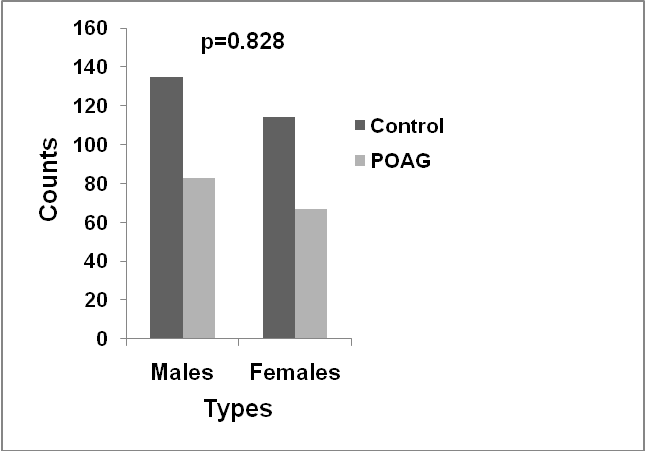

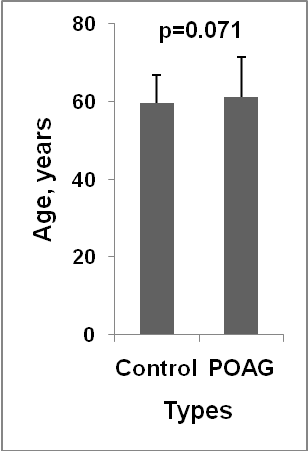


Note: p-value for age was determined by *t*-test and gender distribution by Pearson’s Chi-square analysis.

**Supplementary Table S1**: Association analysis of rs35934224 variant in *TXNRD2* with primary open-angle glaucoma according to gender

| **Group** | **Genetic Model** | **Genotype** | **Control**  **n (%)** | **POAG**  **n (%)** | **Odds ratio (95% confidence interval)** | **p-value** | **p-value*** |
| --- | --- | --- | --- | --- | --- | --- | --- |
| Men | Codominant | C/C | 91 (67.4) | 52 (62.6) | 1.00 | 0.200 | 0.230 |
|  |  | C/T | 41 (30.4) | 25 (30.1) | 1.07 (0.58-1.95) |  |  |
|  |  | T/T | 3 (2.2) | 6 (7.2) | 3.50 (0.84-14.58) |  |  |
|  | Dominant | C/C | 91 (67.4) | 52 (62.6) | 1.00 | 0.470 | 0.600 |
|  |  | C/T-T/T | 44 (32.6) | 31 (37.4) | 1.23 (0.70-2.18) |  |  |
|  | Recessive | C/C-C/T | 132 (97.8) | 77 (92.8) | 1.00 | 0.076 | 0.088 |
|  |  | T/T | 3 (2.2) | 6 (7.2) | 3.43 (0.83-14.10) |  |  |
| Women | Codominant | C/C | 68 (59.6) | 45 (67.2) | 1.00 | 0.120 | 0.110 |
|  |  | C/T | 44 (38.6) | 18 (26.9) | 0.62 (0.32-1.20) |  |  |
|  |  | T/T | 2 (1.8) | 4 (6.0) | 3.02 (0.53-17.20) |  |  |
|  | Dominant | C/C | 68 (59.6) | 45 (67.2) | 1.00 | 0.310 | 0.380 |
|  |  | C/T-T/T | 46 (40.4) | 22 (32.8) | 0.72 (0.38-1.36) |  |  |
|  | Recessive | C/C-C/T | 112 (98.2) | 63 (94.0) | 1.00 | 0.130 | 0.100 |
|  |  | T/T | 2 (1.8) | 4 (6.0) | 3.56 (0.63-19.96) |  |  |

^*^p-value adjusted by age in men and women groups

Abbreviations: POAG, primary open-angle glaucoma

**Supplementary Table S2**: Association analysis of rs6478746 variant near *LMX1B* with primary open-angle glaucoma according to gender

| **Group** | **Genetic Model** | **Genotype** | **Control**  **n (%)** | **POAG**  **n (%)** | **Odds ratio (95% confidence interval)** | **p** | **p^*^** |
| --- | --- | --- | --- | --- | --- | --- | --- |
| Men | Codominant | A/A | 104 (77.0) | 64 (77.1) | 1.00 | 1.000 | 0.990 |
|  |  | A/G | 28 (20.7) | 17 (20.5) | 0.99 (0.50-1.94) |  |  |
|  |  | G/G | 3 (2.2) | 2 (2.4) | 1.08 (0.18-6.66) |  |  |
|  | Dominant | A/A | 104 (77.0) | 64 (77.1) | 1.00 | 0.990 | 0.950 |
|  |  | A/G-G/G | 31 (23.0) | 19 (22.9) | 1.00 (0.52-1.91) |  |  |
|  | Recessive | A/A-A/G | 132 (97.8) | 81 (97.6) | 1.00 | 0.930 | 0.930 |
|  |  | G/G | 3 (2.2) | 2 (2.4) | 1.09 (0.18-6.64) |  |  |
| Women | Codominant | A/A | 91 (79.8) | 53 (79.1) | 1.00 | 0.990 | 0.860 |
|  |  | A/G | 20 (17.5) | 12 (17.9) | 1.03 (0.47-2.27) |  |  |
|  |  | G/G | 3 (2.6) | 2 (3) | 1.14 (0.19-7.07) |  |  |
|  | Dominant | A/A | 91 (79.8) | 53 (79.1) | 1.00 | 0.910 | 0.580 |
|  |  | A/G-G/G | 23 (20.2) | 14 (20.9) | 1.05 (0.50-2.20) |  |  |
|  | Recessive | A/A-A/G | 111 (97.4) | 65 (97.0) | 1.00 | 0.890 | 0.870 |
|  |  | G/G | 3 (2.6) | 2 (3.0) | 1.14 (0.19-6.99) |  |  |

^*^p value adjusted age in men and women groups

Abbreviations: POAG, primary open-angle glaucoma
